# An intranasal selective antisense oligonucleotide impairs lung cyclooxygenase-2 production and improves inflammation, but worsens airway function, in house dust mite sensitive mice

**DOI:** 10.1186/1465-9921-9-72

**Published:** 2008-11-12

**Authors:** Rosa Torres, Aida Herrerias, Mariona Serra-Pagès, Jordi Roca-Ferrer, Laura Pujols, Alberto Marco, César Picado, Fernando de Mora

**Affiliations:** 1 Department of Pneumology and Respiratory Allergy, Hospital Clínic, IDIBAPS, Universitat de Barcelona, Barcelona, Spain; 2 Department of Pharmacology, Universitat Autònoma de Barcelona, Barcelona, Spain; 3 Department of Animal Pathology, Universitat Autònoma de Barcelona, Barcelona, Spain; 4 CIBER (Centro de Investigación Biomédica en Red) de Enfermedades Respiratorias, Spain

## Abstract

**Background:**

Despite its reported pro-inflammatory activity, cyclooxygenase (COX)-2 has been proposed to play a protective role in asthma. Accordingly, COX-2 might be down-regulated in the airway cells of asthmatics. This, together with results of experiments to assess the impact of COX-2 blockade in ovalbumin (OVA)-sensitized mice in vivo, led us to propose a novel experimental approach using house dust mite (HDM)-sensitized mice in which we mimicked altered regulation of COX-2.

**Methods:**

Allergic inflammation was induced in BALBc mice by intranasal exposure to HDM for 10 consecutive days. This model reproduces spontaneous exposure to aeroallergens by asthmatic patients. In order to impair, but not fully block, COX-2 production in the airways, some of the animals received an intranasal antisense oligonucleotide. Lung COX-2 expression and activity were measured along with bronchovascular inflammation, airway reactivity, and prostaglandin production.

**Results:**

We observed impaired COX-2 mRNA and protein expression in the lung tissue of selective oligonucleotide-treated sensitized mice. This was accompanied by diminished production of mPGE synthase and PGE_2 _in the airways. In sensitized mice, the oligonucleotide induced increased airway hyperreactivity (AHR) to methacholine, but a substantially reduced bronchovascular inflammation. Finally, mRNA levels of hPGD synthase remained unchanged.

**Conclusion:**

Intranasal antisense therapy against COX-2 in vivo mimicked the reported impairment of COX-2 regulation in the airway cells of asthmatic patients. This strategy revealed an unexpected novel dual effect: inflammation was improved but AHR worsened. This approach will provide insights into the differential regulation of inflammation and lung function in asthma, and will help identify pharmacological targets within the COX-2/PG system.

## Background

The synthesis of prostaglandins (PG) is catalyzed by either cyclooxygenase (COX)-1 or COX-2, and COX-2 is known to be up-regulated in inflammatory diseases [1]. Although COX-2 and PGs would therefore be expected to be overexpressed in asthma, many observations suggest that this is not always the case. For instance, reports have shown unchanged levels of PGE_2 _in the exhaled breath of asthmatic patients [2], and reduced PGE_2 _and COX-2 levels in smooth muscle cells [3]. Low PGE_2 _production [4] and COX-2 down-regulation [4-7] have been reported in the nasal polyps of asthmatics, in whom the COX-2 up-regulation rate has decreased [6], an observation also inferred from studies in a horse model of asthma [8]. These data suggest that COX-2 may in fact play a protective role in asthma through the production of anti-inflammatory prostanoids [9,10]. This hypothesis is supported by clinical studies in which exogenous PGE_2 _prevented asthmatic responses induced by aspirin, exercise, and allergens [11-13], and by our experiments in house dust mite (HDM)-sensitized mice, in which exogenous PGE_2 _exerted an anti-inflammatory effect [14]. PGI_2 _might also contribute to the anti-asthmatic effects of COX-2 [15], whereas PGD_2 _is mainly considered to favor asthma [16], despite recent evidence to the contrary [17]. It is difficult to account for the defensive properties of COX-2, with results pointing to increased activity of this enzyme in asthma [18-21], but it is likely that the COX-2/PG system functions as a complex network that modulates the asthmatic response according to its fluctuating expression throughout the course of the disease [9]. An accurate understanding of this system could provide novel pharmacological targets [22]. In ovalbumin (OVA)-sensitized mice, the results of the blockade of COX-2 activity provide partial support for a protective role of the enzyme. The number of inflammatory cells in the airway remains unaltered [23] or increases to varying degrees in response to pharmacological inhibition [24-28] or genetic disruption of COX-2 [23,27]. Only Peebles and co-workers and our group [24-26,28] have detected worsening of airway hyperreactivity (AHR). Despite their value, none of the procedures reproduced the reported impaired capacity of asthmatic airways to produce COX-2 [4-7]. Instead, they either induced full blockade (genetic deletion) or reduced activity (inhibitors) of the enzyme. In an attempt to faithfully mimic events in asthmatics, we chose a recently established HDM-induced mouse model of asthma [29], in which we selectively impaired the production of COX-2 in the airways through the use of an antisense oligonucleotide. We then assessed the impact of COX-2 down-regulation on airway inflammation, lung function, and PG production.

## Materials and methods

### Exposure to house dust mite extract

Adult female BALBc mice aged 6 to 8 weeks (Harlan Iberica, Barcelona, Spain) were used in the study. All animal procedures were approved by the Ethics Committee for Animal Research of the Universitat Autònoma de Barcelona.

Sensitization to HDM was induced following a procedure established by Cates et al. [29]. Briefly, the mice were exposed to purified HDM extract (Alk-Abelló, Madrid, Spain) with a very low LPS content (<0.2 EU/dose, measured using the Charles River Endosafe Limulus Amebocyte Assay (Charles River Laboratories, Wilmington, Massachusetts, USA). The allergen was administered intranasally under light halothane anesthesia for 10 consecutive days at a dose of 25 μg/mouse in a 20-μl volume. Non-sensitized (control) animals received intranasal saline.

### Antisense oligonucleotide administration

**Figure 1:**
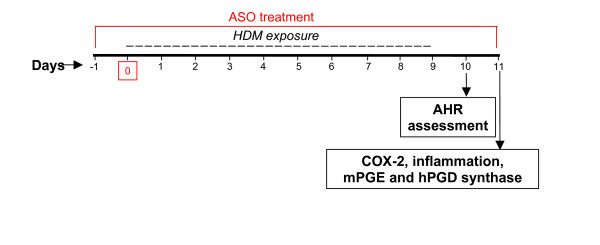
**Sensitization protocol and antisense oligonucleotide (ASO) administration.** Oligonucleotide was administered intranasally one hour before house dust mite (HDM). Twenty-four hours after the last challenge, pulmonary function was assessed by unrestrained whole body plethysmography. Animals were sacrificed the following day and samples were taken.

An antisense oligonucleotide strategy was used to selectively down-regulate the production of lung COX-2 mRNA but not COX-1 mRNA. One day before initiating exposure to HDM, and up to two days after withdrawing the allergen, the mice received intranasal saline (untreated), control mismatched antisense oligonucleotide, or selective COX-2 antisense oligonucleotide at 20 μg/mouse (Figure 1). On the days both products were administered, the treatment was always provided one hour before administering the HDM extract. The COX-2-selective oligonucleotide sequence (IK6 antisense oligonucleotide, 5'GGAGTGGGAGGCACTTGC3') was taken from Khan et al. [30]. The control oligonucleotide contained a 7-base mismatched sequence (5'GGACTAGGTTCAAGTTGC3'). Both oligonucleotides were synthesized with a phosphorothioate backbone to improve resistance to endonucleases. Four experimental groups of mice were therefore established (n = 12 per group): *(1) *untreated non-sensitized, *(2) *untreated HDM-sensitized, *(3) *HDM-sensitized treated with a non-specific control oligonucleotide, and *(4) *HDM-sensitized treated with an antisense oligonucleotide targeting COX-2. For COX-2 mRNA expression, an additional group was included: non-sensitized treated with the COX-2-targeted antisense oligonucleotide (n = 12).

### COX-2 mRNA expression in the lung

COX-2 mRNA expression in the lung was assessed by real time PCR. Total RNA was extracted using Trireagent (Molecular Research Center Inc, Cincinnati, Ohio, USA), and traces of contaminating genomic DNA were removed with DNAfree (Ambion Inc, Austin, Texas, USA). COX-2 cDNA was generated using MMLV reverse transcriptase (Epicentre, Madison, Wisconsin, USA). For real-time PCR, 2 μg of total RNA from each animal was reverse transcribed and the resulting cDNA was placed in the glass capillaries together with 18 μl of a master mix. The COX-2 primers were designed with PrimerSelect software (DNASTAR Inc, Madison, Wisconsin, USA) and were as follows: forward primer, 5'AGCCAGCAAAGCCTAGAGCAACAA3'; and reverse primer, 5'TGACCACGAGAAACGGAACTAAGAGG3'. PCR was performed in a LightCycler instrument and the crossing point (defined as the point at which fluorescence increases appreciably above background fluorescence) was determined with LightCycler software (both from Roche Diagnostics, Mannheim, Germany) using the second derivative maximum method. Using the Relative Expression Software Tool (REST^©^), the relative expression ratio was calculated on the basis of group means for COX-2 as the target gene versus the reference gene GAPDH, and the calculated group ratio was tested for significance using a statistical model known as the Pair Wise Fixed Reallocation Randomization Test^© ^[31]. We took into account the PCR efficiency calculated for COX-2 and GAPDH, which was very similar for both. For purposes of graphical representation, the COX-2 mRNA expression ratio of the non-sensitized untreated mice was established as 1.0, and the average ratios of the other experimental groups were re-calculated on that basis.

### COX-2 protein expression and activity in the lung

**Figure 2:**
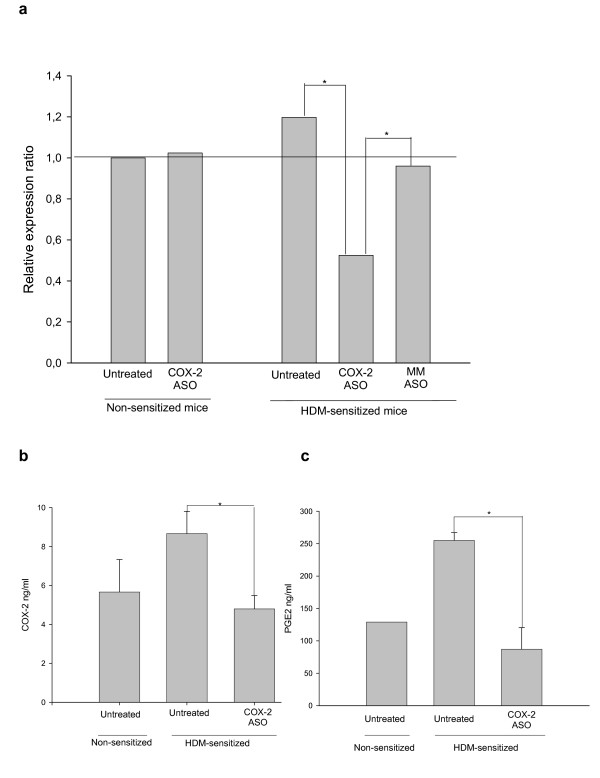
**(a). Relative expression of COX-2 mRNA in lung tissue from different treatment groups assayed by real-time PCR.** The mRNA expression ratio in the non-sensitized mice was established as 1.0. The level of COX-2 mRNA was significantly diminished in the lungs of sensitized mice treated with the selective antisense oligonucleotide (ASO) when compared with both untreated and control oligonucleotide-treated sensitized mice (n = 12). 2 (b). Levels of COX-2 protein in the lung tissue of non-sensitized mice (n = 3) and in untreated and COX-2 ASO-treated HDM-sensitized mice (n = 6). 2 (c) PGE_2 _content in the bronchoalveolar lavage (BAL) of non-sensitized (n = 1) and HDM-sensitized treated (n = 4) and untreated (n = 3) mice (* *p *< 0.05). MM, mismatched oligonucleotide.

To support lung COX-2 mRNA assessment, the enzyme's airway protein expression and activity were determined in some of the mice from each experimental group (from 3 to 6 mice see legend of Figure 2b and 2c). Briefly, proteins were extracted from the right lung lobe of each animal using a lysis buffer containing protease inhibitors (Mini complete tablet, Roche Diagnostics, Mannheim, Germany). The concentration of COX-2 protein was determined by ELISA (IBL, Hamburg, Germany).

Immunohistochemistry for COX-2 was performed in lung sections using a polyclonal antibody (sc-1746, Santa Cruz Biotechnology, Santa Cruz, California, USA) after boiling in 10 mM citrate buffer (pH 6) to retrieve the antigen. The sections were then incubated overnight at 4°C with or without the primary antibody. A rabbit anti-goat secondary antibody (Vector, Burlingame, California, USA) was used at room temperature for 1 hour, followed by incubation with horseradish peroxidase-conjugated avidin-biotin complex (Pierce, Rockford, Illinois, USA). Staining was then performed with diaminobenzidine to reveal immunolabeling. Additionally, prostanoids were extracted from BAL fluid and purified through Sep-Pak C_18 _columns (Waters Corporation, Milford, Massachusetts, USA). After evaporation and resuspension in EIA buffer, the PGE_2 _concentration was measured using a commercially available specific ELISA (Cayman Europe, Tallin, Estonia).

### Pulmonary function testing

To assess the effect of impaired COX-2 production on airway function, we analyzed the in vivo airway reactivity to increasing doses of nebulized methacholine (6.25 to 100 mg/ml) 24 hours after the last exposure to HDM in either treated or untreated sensitized and non-sensitized mice (Figure 1), using a well established [32-34] non-invasive whole-body plethysmography (WBP) technique (Buxco Europe Ltd, Winchester, UK), which is based on changes in the time elapsed between nasal and thoracic pressure fluctuations. The response to methacholine was averaged and expressed as Penh (Enhanced Pause) as previously reported [14]. Results were compared by two-way ANOVA. In the BALB/c mice strain, Penh has been shown to correlate with lung mechanics (R_L_) [35]. This can also be inferred from recent studies, in which AHR was assessed using WBP in combination with invasive procedures [36,37].

### Assessment of airway inflammation

Inflammation was evaluated 48 hours after the last exposure to HDM (Figure 1) using two approaches. Total cellularity in BAL samples was counted immediately after fluid collection. BAL was performed by slowly infusing 0.3 ml of phosphate-buffered saline (PBS) (2% fetal bovine serum) twice and recovering it by gentle aspiration 30 seconds after delivery. A 20-μl aliquot of BAL fluid was stained with Turk solution (0.01% crystal violet in 3% acetic acid) and analyzed in a Neubauer chamber. In addition, an experienced blinded observer counted the number of Congo red-stained eosinophils twice in histological lung sections of the same animals. The tissue area was also measured using MIP 45 Advanced System image analysis software (Microm España, Barcelona, Spain) to express the number of eosinophils per mm^2^. Group means (n = 12) were compared using an unpaired *t *test.

### Expression of mPGE synthase and hPGD synthase in the lung

The expression of mPGE synthase and hPGD synthase was determined by conventional reverse transcriptase-PCR using previously described primer pairs [38] in mRNA extracted from the lungs. GAPDH was assessed as the reference gene. Samples were denatured for 5 min at 95°C, and the cycling parameters (35 cycles) for mPGES, hPGDS, and GAPDH were 95°C for 30 sec, 55°C for 30 sec, and 72°C for 30 sec. A final extension step of 8 min at 72°C was applied. Amplification products were separated by agarose gel electrophoresis. After staining with ethidium bromide, the optical density of the bands was analyzed using Quantity One software (Bio-Rad Laboratories, Hercules, California, USA). The ratios of the band densities were calculated for each enzyme versus GAPDH and the means (n = 12) were compared using an unpaired *t *test.

### Statistical analysis

Data in the text, table, and figures are expressed as the mean ± standard error of the mean (SEM) unless otherwise stated. Differences in airway response between different groups were tested for statistical significance using ANOVA followed by a post hoc Bonferroni test. The unpaired *t *test was used for all other analyses. Differences were considered statistically significant when the probability value was less than 0.05.

## Results

### Intranasal antisense oligonucleotide impairs pulmonary COX-2 expression and activity

Figure 2a shows the relative expression ratio of COX-2 mRNA in lung tissue, where 1.0 was established as the baseline level in the untreated non-sensitized mice. The level in the lung tissue of HDM-sensitized mice treated with the control oligonucleotide was the same as in the lung tissue of the untreated sensitized mice. In contrast, COX-2 mRNA expression was significantly diminished in sensitized mice treated with the COX-2-specific antisense oligonucleotide compared with untreated (55% decrease) or mismatched control oligonucleotide-treated HDM-sensitized mice. In the non-sensitized mice's airway, the antisense oligonucleotide did not exert any effect on COX-2 mRNA expression. Our data also revealed that there were no statistically significant differences in the expression of lung COX-2 between non-sensitized and HDM-sensitized mice.

**Figure 3:**
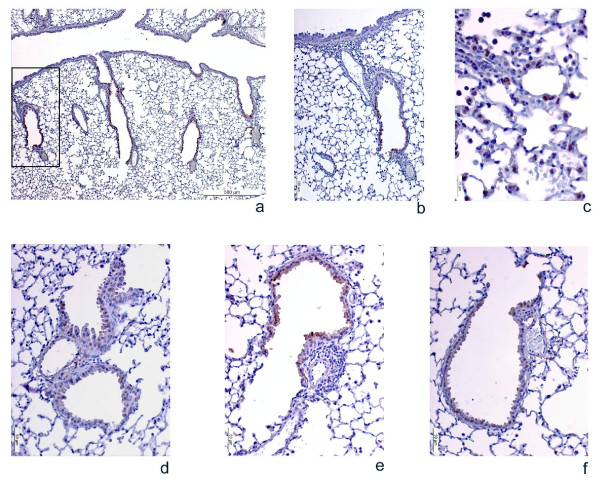
**Photomicrographs of COX-2 immunolabeling in lung samples from HDM-sensitized mice following different treatments.** Pictures *a*, *b*, and *c *show representative images of the COX-2 immunostaining pattern in the airways. Since the COX-2 distribution was almost the same in all 4 experimental groups, only representative images of one of them are included. 3 (a) shows a general view of COX-2 distribution in the airways, where labeling is detected in the bronchiolar epithelium but not in the principal airway. 3 (b) shows a single bronchiole (magnified view of the area outlined in [a]), and 3 (c) shows stained alveolar macrophages. Pictures d, e, and f reflect the consistent changes in the COX-2 antigen signal intensity under different experimental conditions. 3 (d) shows a single bronchiole from a non-sensitized mouse, 3 (e) A single bronchiole from a sensitized mouse treated with control mismatched oligonucleotides (MM), and 3 (f) COX-2 protein expression in the airways after treatment with the COX-2 antisense oligonucleotide (ASO). Similar staining intensity was seen in untreated sensitized mice, in which cells were heterogeneously labeled and peribronchial and perivascular inflammation was observed, but the immunostaining signal diminished clearly and consistently in the treatment group.

The concentration of COX-2 in lung protein extracts from some of the mice was measured by ELISA. A significant 45% reduction in COX-2 protein concentrations was observed in the lungs of COX-2 antisense-treated sensitized mice compared with non-treated sensitized mice (Figure 2b). Analysis of COX-2 expression in the lung by immunohistochemistry showed the same results for untreated HDM-sensitized mice and mismatched control oligonucleotide-treated sensitized mice (Figure 3e), that is, they consistently had a visibly increased number of positive cells and a stronger staining intensity than selective COX-2 oligonucleotide-treated mice (Figure 3f).

The photomicrographs in Figure 3 also show that the pattern of COX-2 expression in the airways of the mice was restricted to secondary and tertiary bronchi and bronchiolar epithelial cells (Figure 3a and 3b), as well as alveolar macrophages (Figure 3c). No expression whatsoever was observed in the epithelium of the main bronchi in any of the animals studied. This distribution pattern of COX-2 protein was found to be identical in all the experimental groups, regardless of whether they were sensitized or not, or treated or not. Only representative pictures of the pattern are included.

Finally, the PGE_2 _concentration in BAL fluid was significantly (66%) lower in the lungs of COX-2 antisense oligonucleotide-treated sensitized mice than in the untreated sensitized ones (Figure 2c). Thus, impairment of COX-2 expression was accompanied by a similar level of impaired activity.

### Selective impairment of pulmonary COX-2 increases airway hyperreactivity

**Figure 4:**
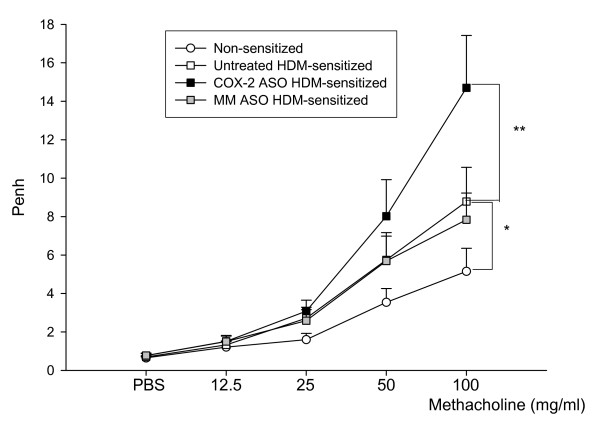
**Airway reactivity to increasing concentrations of aerosolized methacholine.** Airway reactivity is shown in non-sensitized mice (white circles), untreated sensitized mice (white squares), control mismatched oligonucleotide-treated sensitized mice (grey squares), and selective COX-2 antisense sensitized mice (black squares). Two-way analysis of variance was used to compare the curves. The COX-2 antisense oligonucleotide (ASO)-treated mice showed a significant increase in AHR to methacholine compared with both untreated and control oligonucleotide-treated sensitized mice. Data are shown as the mean ± SEM (**p < 0.01, ***p < 0.005). ASO: antisense oligonucleotide, MM: mismatched oligonucleotide (n = 12).

Increasing doses of methacholine induced a dose-dependent rise in Penh in all experimental groups (Figure 4). The Penh increase was higher in the HDM-sensitized mice than in the non-sensitized mice, revealing the induction of AHR in the HDM-sensitized animals. As shown in Figure 4, untreated and control oligonucleotide-treated sensitized mice had almost identical responses to the bronchoconstrictor. However, AHR was significantly higher in the selective COX-2 oligonucleotide-treated mice, where the Penh value at the maximum methacholine concentration (100 mg/ml) was almost twice that found in the untreated or mismatched control-treated sensitized animals (7.40 ± 1.62 vs 14.12 ± 2.75).

### Selective impairment of pulmonary COX-2 reduces airway inflammation

**Figure 5:**
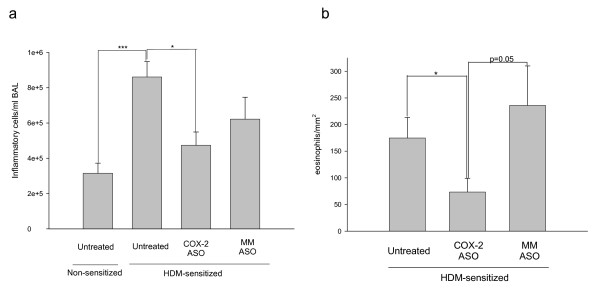
**Airway inflammation in non-sensitized and in untreated or treated HDM-sensitized mice.** Graph (a) shows the total inflammatory cell count in bronchoalveolar lavage fluid, and graph (b) depicts the eosinophils infiltrating the airways in the same animals. In both cases, the selective COX-2 antisense oligonucleotide caused a significant reduction in the accumulation of inflammatory cells in the lungs. No differences were found between the untreated and the control mismatched oligonucleotide-treated sensitized mice. Data are shown as means ± SEM (*p < 0.05, **p < 0.01). ASO: antisense oligonucleotide, MM: mismatched oligonucleotide (n = 12)

HDM-sensitized mice developed a clear peribronchial and perivascular eosinophilic inflammation (Figure 3e) with goblet cell hyperplasia, compared with the non-sensitized animals. No differences were found in the total number of inflammatory cells or in eosinophil accumulation between untreated and mismatched control oligonucleotide-treated sensitized animals (Figure 5a and 5b). In contrast, when COX-2 was selectively inhibited in sensitized mice, the level of inflammation was clearly and significantly reduced to 50%–55% of its value in the untreated HDM-sensitized mice, depending on whether total cells or eosinophils were considered (Figure 5a and 5b, respectively). This reduced inflammation was also visible in the lung sections (Figure 3f).

### Selective blockade of pulmonary COX-2 inhibits mPGE2 synthase but not hPGD2 synthase

The mRNA expression of mPGE_2 _and hPGD_2 _was normalized to the level of constitutive GAPDH (Table 1). No differences were observed between the non-sensitized and the untreated sensitized mice. The expression ratio in the untreated and the mismatched control oligonucleotide-treated mice was also the same for both enzymes. Likewise, no differences were found in the mRNA expression of hPGD_2 _synthase in the lungs of mice from any of the experimental groups, whether or not they were treated with COX-2 antisense oligonucleotide. In contrast, HDM-sensitized mice treated with the selective COX-2 oligonucleotide displayed significantly reduced expression of mPGE synthase (40% reduction) compared with mismatched control oligonucleotide-treated or untreated sensitized animals.

**Table 1:** Expression ratio of mPGE synthase and hPGD synthase mRNA in lung tissue from different treatment groups^a^

Expression ratio	Non-sensitized	Untreated HDM-sensitized	MM ASO HDM-sensitized	COX-2 ASO HDM-sensitized
mPGE synthase/GAPDH	1.105 ± 0.09	1.047 ± 0.09	1.068 ± 0.13	0.624 ± 0.13*
hPGD synthase/GAPDH	0.369 ± 0.06	0.417 ± 0.06	0.335 ± 0.05	0.365 ± 0.06

^a ^mRNA expression was analyzed by conventional reverse transcriptase-polymerase chain reaction and the expression ratios compared with GAPDH were determined by densitometry. No differences were observed in hPGD synthase mRNA expression, either between non-sensitized and sensitized animals or between HDM-sensitized animals treated with mismatched antisense oligonucleotides and animals treated with COX-2 antisense oligonucleotide. In contrast, mPGE synthase expression was reduced in the selective COX-2 oligonucleotide-treated mice compared with the other experimental groups. Data are shown as the mean ± SEM (*p< 0.05). ASO, antisense oligonucleotide; MM, mismatched oligonucleotide (n = 12).

## Discussion

This study shows that intranasal administration of a selective COX-2 antisense oligonucleotide impairs COX-2 expression and activity in the lungs, and that this change has a fairly unusual effect on the airway response of HDM-sensitized mice, namely, AHR worsens while airway inflammation improves. This COX-2-driven modulation is associated with reduced ability of airway cells to synthesize PGE_2_, but apparently not PGD_2_, as might be deduced from the expression of the corresponding PG synthases.

To our knowledge, this is the first study in which COX production has been impaired using antisense technology in a murine model of asthma. Rather than induce complete inhibition of COX-2 production or impair its activity, the inhibitory strategy used in our experiment was intended to mimic the described reduced production of COX-2 [5-7] and hence PG [4,8,39] in asthma. Since there were no previous records on the ideal conditions of intranasal administration of the anti-COX-2 oligonucleotide, we chose the most efficient sequence used in another biological system in which it had been optimized to guarantee an effect [30]. Our goal was to ensure efficient down-regulation of COX-2 mRNA during the relevant phases of sensitization and challenge. In contrast to systemic inhibition of the enzyme [23-27], in our experiment the blocking agent was delivered within the airway, a strategy that most likely restricts its effects to the lungs [40]. Finally, the use of a natural aeroallergen, and its daily administration exclusively through the airways, allows an accurate reproduction of the exposure in humans. Therefore we feel that ours is an extremely suitable approach to determine the role of COX-2 during the course of asthma.

To validate our inhibitory strategy, we analyzed lung COX-2 mRNA expression. Furthermore, the COX-2 mRNA data were supported by the analysis of airway COX-2 protein expression and the assessment of its activity by measuring the production of one of its main products, PGE_2 _[41]. Irrespective of the variable considered, the degree of inhibition of airway COX-2 achieved with antisense oligonucleotide treatment was between 45% and 66%. Although immunohistochemistry is not a quantitative technique, we could consistently identify a stronger COX-2 antigen signal in the airway of mice not receiving the antisense oligonucleotide. In addition, since immunohistochemistry revealed that COX-2 expression was consistently restricted to the bronchial epithelium of the lower airways, we can be fairly certain that the method used to deliver the oligonucleotide reached deep areas of the bronchial tree. This is not surprising, since intranasal provision of siRNA targeting other molecules has been shown to affect the alveoli [42]. The restricted expression of COX-2 in the lower airways of non-asthmatic animals is consistent with a previous observation in healthy mice [43].

Interestingly, lung COX-2 mRNA from non-sensitized mice was not affected by the antisense oligonucleotide, suggesting a differential effect of the oligonucleotide in allergen-sensitized and non-sensitized scenarios, a hypothesis that would require specific validation. Accordingly, it is noteworthy that, although COX-2 expression was not significantly increased in sensitized versus non-sensitized mice, there was a trend towards up-regulation. However, if any, the overproduction of COX-2 is mild in the HDM-sensitized animals. Although these results appear to contradict previous data [18-21], they are plausible in the light of other observations in patients in whom exhaled PGE_2 _remains unchanged [2], and in whom COX-2 production may even be impaired [3-7]. These discrepancies favor the hypothesis of fluctuating enzyme activity during the course of the disease [9], as suggested in other models [44].

The impairment of COX-2 expression and activity caused by the antisense oligonucleotide was associated with a worsening of AHR. Pulmonary function was evaluated using WBP, a method that has been shown to correlate with lung resistance in BALB/c mice [35], and whose validity is endorsed by recent publications [36,37,45], although it has also come under criticism [46]. Therefore, COX-2 products appear to limit the HDM-induced AHR and play a protective role at the airway functional level. The resulting reduced production of endogenous PGE_2 _and mPGE synthase may be directly linked to the observed worsening of AHR according to previous observations [13]. Using an alternative experimental approach, Peebles et al. [26] showed an IL-13-dependent increased AHR under COX-2 inhibition in OVA-sensitized mice. We presume that this Th_2 _cytokine might at least partly explain our increased AHR [29], but other elements, such as cys-leukotrienes, which presumably are increased when the COX pathway is partly blocked, should not be ruled out as directly responsible for the in vivo worsening of airway function in the presence of antisense oligonucleotide targeting COX-2 [47,48].

Our data contrast with the findings of other groups [23,27]. The differences are probably attributable to methodological issues, including their use of a full COX-2 blockade (gene knockout strategy) compared with our partial blockade through interference with transcriptional events.

We would have expected the worsening of AHR to be paralleled by increased airway inflammation. However, eosinophilic inflammation in COX-2 antisense oligonucleotide-treated sensitized mice fell to 50% of the inflammatory burden in untreated HDM-sensitized mice. Thus, under our conditions, COX-2 products appear to enhance proinflammatory signals. PGE_2 _could also be responsible for such an effect, since in vitro and ex vivo data suggest that this PG contributes to migration of mast cells and dendritic cells and, therefore, promotes inflammation [49,50]. However, we believe that a more complex mechanism involving more than one agent contributes to such a beneficial antisense oligonucleotide-driven effect. It has been shown in a rat model of acute lung injury that inflammation worsened when COX-2 was down-regulated [51]. The opposing actions of COX-2 transcription impairment on AHR and inflammation are difficult to interpret. There is a general belief that AHR at least partly correlates with the underlying inflammatory process, since respiratory dysfunction is normally accompanied by airway inflammation in humans and in murine models. Even though reports have been published in which AHR and inflammation did not fluctuate in parallel [23,52,53], to our knowledge, an inverse correlation of the magnitude seen here has not been previously reported. A change in airway smooth muscle reactivity with no underlying changes in airway inflammation, or vice versa, can certainly be interpreted as the result of different mechanisms leading to two dissociated phenomena. However, our data raise a different hypothesis; AHR and inflammation can be differentially modulated to the extent that impaired COX-2 production leads to a negative correlation between them, and this therefore raises the possibility of an inverse association of both phenomena, rather than a dissociation. The use of an antisense oligonucleotide targeted to COX-2 administered intranasally in HDM-sensitized mice has uncovered a key element in establishing the mechanisms involved in COX-PG-controlled alteration of the asthma response.

Finally, it is noteworthy that our unchanged levels of hPGD synthase suggest that PGD_2 _production was probably unaffected [54], and, therefore, that the impact of this PG on the oligonucleotide-induced changes was limited. Despite the fact that PGD_2 _is usually considered relevant immediately after challenge [16,55], we measured the synthase 48 hours after the last exposure to the allergen, a factor that may explain our negative results.

## Conclusion

Administration of antisense oligonucleotides provides a fairly accurate way to target a single molecule within the airway environment while minimizing unwanted systemic effects. This interesting model allows us to address the potentially inadequate regulation of COX-2 in asthmatic patients. Although our data confirm the protective effect attributable to COX-2 products in relation to airway function, they also highlight a role for COX-2 in the generation of proinflammatory signals. How and when these opposing functions occur should be the focus of future research to identify potential pharmacological targets in the COX-2/PG system.

## Competing interests

The authors declare that they have no competing interests.

## Authors' contributions

FDM obtained funding for the project, provided overall guidance for the study, assisted in the analysis and interpretation of the data, and prepared the manuscript. RT participated in the experimental design, planned and performed all of the experiments, and helped in the writing of the manuscript. AH, AM, MS, LP, and JRF participated in sample and data collection and helped in the revision of the manuscript. CP participated in the acquisition of funding, designing the experiments, and revising the manuscript. All the authors have read and approved the final manuscript.
